# Bone Mineral Density at Diagnosis of Celiac Disease and after 1 Year of Gluten-Free Diet

**DOI:** 10.1155/2014/173082

**Published:** 2014-10-14

**Authors:** Stefano Pantaleoni, Massimo Luchino, Alessandro Adriani, Rinaldo Pellicano, Davide Stradella, Davide Giuseppe Ribaldone, Nicoletta Sapone, Gian Carlo Isaia, Marco Di Stefano, Marco Astegiano

**Affiliations:** ^1^Department of Gastroenterology, Molinette Hospital, University of Turin, 10126 Turin, Italy; ^2^Clinic of Gastroenterology and Hepatology, Via Cavour 31, 10123 Turin, Italy; ^3^Gerontology and Bone Metabolic Disease Section, Molinette Hospital, University of Turin, 10126 Turin, Italy

## Abstract

Atypical or silent celiac disease may go undiagnosed for many years and can frequently lead to loss of bone mineral density, with evolution to osteopenia or osteoporosis. The prevalence of the latter conditions, in case of new diagnosis of celiac disease, has been evaluated in many studies but, due to the variability of epidemiologic data and patient features, the results are contradictory. The aim of this study was to evaluate bone mineral density by dual-energy X-ray absorptiometry in 175 consecutive celiac patients at time of diagnosis (169 per-protocol, 23 males, 146 females; average age 38.9 years). Dual-energy X-ray absorptiometry was repeated after 1 year of gluten-free diet in those with *T*-score value <−1 at diagnosis. Stratification of patients according to sex and age showed a higher prevalence of low bone mineral density in men older than 30 years and in women of all ages. A 1-year gluten-free diet led to a significant improvement in lumbar spine and femoral neck mean *T*-score value. We propose that dual-energy X-ray absorptiometry should be performed at diagnosis of celiac disease in all women and in male aged >30 years, taking into account each risk factor in single patients.

## 1. Introduction

Celiac disease (CD) is a chronic immune-mediated disorder, characterized by villus atrophy of the proximal small intestinal mucosa and malabsorption of nutrients after the ingestion of wheat gluten or related proteinsin genetically susceptible individuals expressing the HLA class II molecules DQ2 or DQ8. Prompt clinical and histologic improvement is observed following strict adherence to a gluten-free diet (GFD) [1]. Several extraintestinal manifestations, including anemia, osteopenia, neurologic symptoms, menstrual abnormalities, infertility, recurrent spontaneous abortions, growth retardation, dermatitis herpetiformis, aphthous stomatitis, and dental defects, have been associated to CD [2].

In the last decades, screening studies have shown a higher prevalence of CD than previously thought; up to 1% of the European and US population are affected at any age and in a wide variety of clinical circumstances [3, 4]. In the past, CD was almost always recognized because of its classical presentation, seen mainly in children, characterized by a predominance of digestive symptoms like diarrhea, weight loss, and growth retardation. Today, the presentation of CD is more frequently atypical, with confusing symptoms or without symptoms at all. These latter forms may remain undiagnosed for many years and can lead, among various consequences, to loss of bone mineral density (BMD) [5]. Since gastrointestinal symptoms, associated disorders, and complications can be prevented by adherence to dietary therapy, early recognition of CD is crucial.

Osteoporosis is characterized by severe BMD loss leading to enhanced bone fragility, and, consequently, atraumatic fractures. Although this disease can involve any bone, the hip, spine, and wrist are most likely to be affected with a remarkable burden for the Public Health System [6]. Malabsorption syndromes, calcium deficiency, and corticosteroids administration are well-known causes of secondary osteoporosis [7]. Dual-energy X-ray absorptiometry (DXA) at the femoral neck and lumbar spine is considered the gold standard to confirm the diagnosis of osteoporosis [8]. Moreover, DXA is one of parameters of FRAX, a diagnostic tool used to evaluate the 10-year probability of bone fracture risk [9].

BMD in celiac patients has been evaluated in many studies but, due to their design, there are several discrepancies regarding the prevalence of osteoporosis/osteopenia at CD diagnosis [10, 11]. Since the use of DXA is not justified in all CD patients, it is crucial to select the population appropriate for this test [12]. The aim of this study has been to evaluate the prevalence of abnormalities at DXA (osteopenia/osteoporosis) in patients with new diagnosis of CD and to assess the impact of GFD on BMD 1 year after the diagnosis.

## 2. Materials and Methods

### 2.1. Patients

We conducted a prospective study, consisting of evaluation of BMD using DXA on 175 consecutive outpatients with new diagnosis of CD and after 1 year of GFD. Renal function was normal and none of these patients were on treatment with calcium or vitamin D.

**Figure 1:**
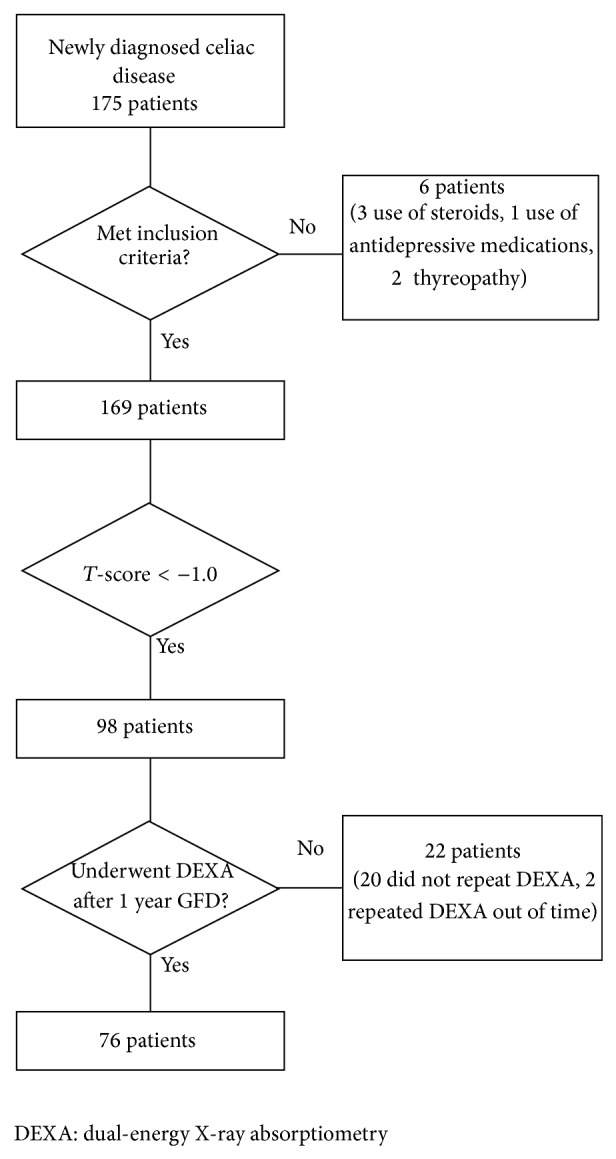
Patients included in the study.

CD diagnosis was defined using the following criteria indicated by International guidelines: (a) positive immunoglobulin (IgA) anti-tissue transglutaminase antibody (tTG) titers or anti-endomysial antibodies (EmA) [13]; in case of IgA deficiency, IgG anti-tTG titer was determined; (c) six biopsy specimens were taken from second part of duodenum during the endoscopic examination; the degree of mucosal damage was scored according to the Marsh-Oberhuber classification [14]; for the purpose of the study, only patients who had classical duodenal mucosal villus atrophy and crypt hyperplasia (Marsh grade III) were considered. To guarantee a greater uniformity, the same expert pathologist analyzed all the samples. Exclusion criteria included patients with previous diagnosis of CD, use of immunosuppressive medication (steroids, tacrolimus), heparin, antidepressive agents, surgical menopause, hormonal and metabolic disorders known as cause of low BMD, and patients with thyreopathy, liver, or kidney diseases (Figure 1). No patient had history of malabsorption due to inflammatory bowel disease (IBD) or previous intestinal resections.

Before beginning GFD, the BMD of the lumbar spine and femoral neck was measured by DXA scanning (Hologic; Bedford-MA) at Molinette Hospital, Turin, Italy. According to WHO criteria, a* T*-score of ≥−1 denotes normal bone, a* T*-score between −1 and −2.5 denotes osteopenia, and a* T*-score of ≤−2.5 denotes osteoporosis (*Z*-score alone is not used to diagnose osteoporosis in premenopausal women) [15].

After 1 year of GFD (12 ± 1 month) only patients that showed a* T*-score value <−1 at diagnosis repeated DXA. Patients who refused to repeat DXA were excluded from the final analysis. During this year, neither calcium nor vitamin D was given to patients. Patients with nonresponsive celiac disease (NRCD) (positive IgA anti-tTG or anti-EmA, in case of IgA deficiency IgG anti-tTG) after 1 year of GFD were excluded from the final analysis (only in these patients, excluded from the final analysis, upper endoscopy with intestinal biopsies was repeated, according to current guidelines [13]).

### 2.2. Statistical Methods

Statistical analyses were conducted using Med Calc version 9.2.1.0 software. The signed rank sum test (Mann-Whitney test) (independent samples) has been used to compare different patient groups because size of groups is different and paired samples *t*-test was used to compare the same group of patients at different times. All analyses were two tailed and *P* value < 0.05 was considered significant.

The study was conducted in accordance with ICH-Good Clinical Practice guidelines, the Declaration of Helsinki, and local laws and regulations. The protocol was approved by the appropriate independent ethics committees, and patients gave written informed consent.

## 3. Results and Discussion

A total of 169 (23 males, 146 females) outpatients were considered. The average age was 38.9 years (range 17–75) (Table 1).

According to WHO criteria, at the time of diagnosis, a normal DXA at lumbar spine was observed in 71 patients (42%;* T*-score mean value −0.1 ± 0.7), whilst 62 were osteopenic (37%;* T*-score mean value −1.7 ± 0.4) and 36 osteoporotic (21%;* T*-score mean value −3.1 ± 0.4) with a total population mean* T*-score value of −1.3 ± 1.3. A normal DXA at femoral neck was observed in 72 patients (43%; mean value −0.3 ± 0.6), whilst 75 were osteopenic (44%; mean value −1.7 ± 0.4) and 22 osteoporotic (13%; mean value −3.1 ± 0.4) with a total population mean* T*-score value of −1.3 ± 1.1. There was no statistically significant difference in mean* T*-score values, neither at lumbar spine (*P* = 0.43) nor at femoral neck (*P* = 0.48), between females and males, but it is worth noting that there was a difference of 0.2/0.3 standard deviation (s.d.) among these groups.

The subdivision of the study population by age and sex groups (Table 2) showed a higher prevalence of low BMD at lumbar spine in men older than 30 years and women of all ages.

There was no statistically significant difference (*P* > 0.05) between the different age groups of males. Contrariwise, in females there was a significant difference (*P* < 0.0001) between lumbar* T*-score between over 50 years group and less than 30 years group (the same for femoral* T*-scores).

No statistically significant difference was found between Marsh IIIA e Marsh IIIC (*P* = 0.27) in lumbar spine* T*-score values as between Marsh IIIA (partial villus atrophy) and Marsh IIIC (total villus atrophy) (*P* = 0.089) in femoral neck* T*-score values.

### 3.1. Patients after 1-Year Gluten-Free Diet

**Figure 2:**
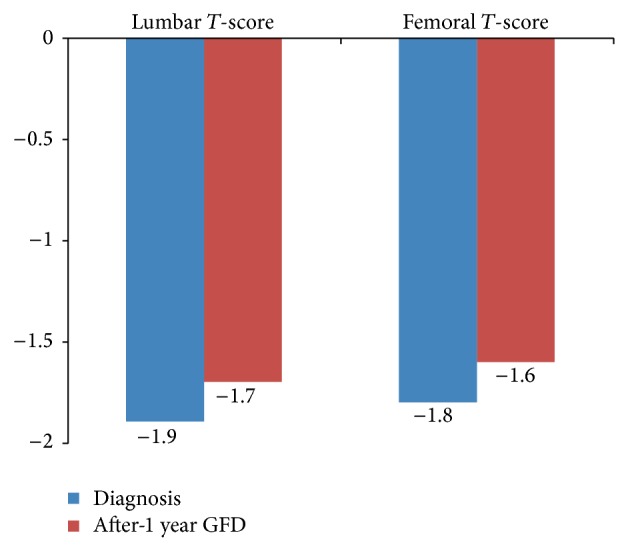
Bone mineral density (BMD) score improvement after 1-year GFD in total population.

A 1-year GFD led to a significant improvement in lumbar spine and femoral neck mean* T*-score value (from −1.9 ± 1.2 to −1.7 ± 1.3, *P* = 0.015, and from −1.8 ± 1.0 to −1.6 to 1.0, *P* < 0.001, resp.) in the 76 patients (31 postmenopausal women, 36 postmenopausal women, and 9 men) who underwent a reassessment of BMD (Figure 2).

**Figure 3:**
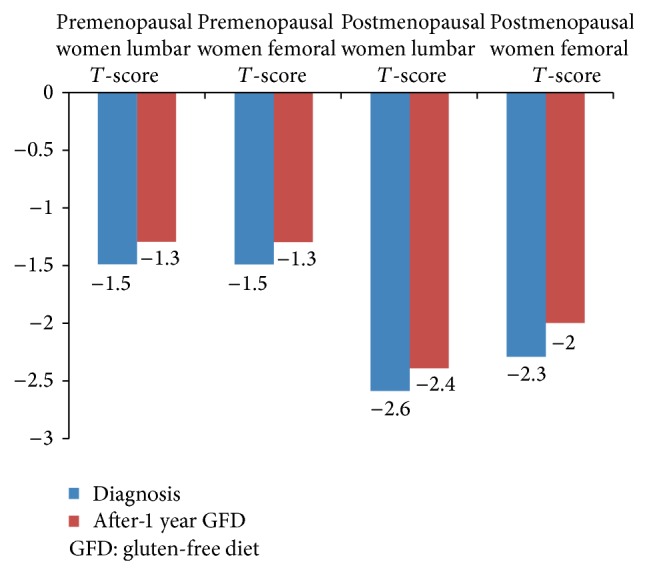
Bone mineral density (BMD) score improvement after 1-year GFD according to menopausal status.

In premenopausal women, GFD induced a significant improvement in femoral* T*-score (*P* = 0.02) but not in lumbar* T*-score (*P* = 0.064). The same results were observed for postmenopausal women, with a significant improvement in femoral* T*-score (*P* = 0.01) but not in lumbar* T*-score (*P* = 0.22). Overall, pre/postmenopausal women's data are reported in Figure 3. Due to the small number of men in the study, it is impossible to draw any conclusions on improvement in men.

## 4. Discussion

In adults, bone remodeling has several functions: to repair microdamage within the skeleton, to maintain skeletal strength, and to supply calcium from the skeleton to maintain serum calcium levels. To better understand the fine details of these events, several pathways of osteogenic induction are under scrutiny [16]. The alterations of these functions, due to several causes, can lead to osteopenia or osteoporosis. While an inflammatory pattern is more plausible in the pathogenesis of osteoporosis occurring in patients with IBD [17], a combination of malabsorption and inflammation contributes to low BMD in CD patients. Intestinal mucosal lesion can lead to calcium malabsorption and decreased levels of serum calcium [18–20] along with hypovitaminosis D [20, 21]. Chronic release of proinflammatory cytokines by immunologically competent cells of the gastrointestinal mucosa might induce bone remodeling, stimulating bone resorption by osteoclasts [22].

This single-center, prospective study investigates the prevalence of low BMD and the effects of 1-year gluten withdrawal in a cohort of adult patients with new diagnosis of CD.

The prevalence of low BMD (osteoporosis and osteopenia) at the moment of CD diagnosis was 58%, but it is not easy to compare our data with those available in the literature [18, 23–25] due to the variability of characteristics (age, sex, Marsh stage, and pre- and postmenopausal status) of the individuals included in those studies. Nevertheless, it is important to identify the patients who are most at risk of fractures, to evaluate the different possibilities of surveillance and treatment. In the present study, females/males ratio was higher compared to other reports [26]. A large number of males were excluded because of their refusal to repeat DXA 1 year after the diagnosis or repeated DXA out of date. In fact, the proportion of males that repeated DXA 1 year after CD diagnosis was only 12%. In our cohort, there was no significant difference in lumbar/femoral* T*-score mean values between males and females. BMD scores according to Marsh III stage did not show any statistically significant difference. García-Manzanares et al. [27] found that the stage of duodenal mucosal injury (following Marsh classification) was the most important factor in determining low BMD at diagnosis but they considered differences between Marsh I, Marsh II, and Marsh III grades unlike our study design.

A higher prevalence of low BMD was observed in men older than 30 years and women of all ages, indicating that these groups should be considered candidates for DXA scan at the moment of CD diagnosis. Furthermore, our study shows that 1 year of dietary treatment led to a bone mass improvement as reported in other studies [23–25, 28, 29].

Finally, as the main consequence of osteoporosis is the increased fracture risk, and the latter in these individuals seems to be modest [12], performing DXA routinely in all newly diagnosed CD patients cannot be considered. It is more appropriate to select those patients who are at higher risk of fractures for DXA. Thus, although some authors proposed that a screening should be performed in all cases, either at the time of diagnosis or after 1 year of GFD [12, 30, 31], we believe that the modest increase in fracture risk does not justify the costs of such screening [32].

Some critical issues should be considered. The retrospective design of the study represents a limitation: in fact, in real life, control biopsies after 1 year of GFD are performed only in NRCD, while control biopsies would be useful to check if there is a correlation between a lack of improvement of BMD after one year of GFD and a lack of complete normalization of duodenal lesions despite symptoms disappearance and negative CD related serology [33].

In the literature, prevalence of osteoporosis in celiac disease is increased at the lumbar spine, but it is uncommon at femoral neck; axial bone mass increases more than appendicular mass during GFD therapy [34]. In our study DXA scan of the lumbar spine resulted in osteoporosis in 21% of patients, while DXA scan of the femoral neck resulted in osteoporosis in 13% of patients; a 1-year GFD led to a significant improvement in lumbar spine and femoral neck mean* T*-score value (*P* = 0.015 and *P* < 0.001, resp.).

The improvement in BMD occurs mostly within the first year; some patients diagnosed in adulthood run the risk of maintaining a low BMD. Since BMD improves after 1 year of GFD [35], it would be reasonable to repeat DXA at this time and to start a supplementation therapy with calcium and vitamin D only in patients without an improvement of BMD after the GFD.

## 5. Conclusions

In conclusion, we propose to perform DXA at diagnosis of CD in all women and in men older than 30 years, considering in each patient all the potential risk factors (age, prior osteoporotic fracture, family history of hip fracture, low body mass index, use of drugs like corticosteroids, smoking, and alcohol excess). Long-term studies are however needed to evaluate the contribution of these factors to risk of fractures in CD patients.

## Figures and Tables

**Table 1:** Baseline characteristics of study population.

Celiac disease (*N*)	169
Female (*N*, %)	146, 86.4%
Male (*N*, %)	23, 13.6%
F/M ratio	6.3 : 1
Average age (years, SD)	38.9 ± 12.6
Premenopausal female (*N*, %)	104, 61.5%
Postmenopausal female (*N*, %)	42, 24.9%

*N*; number.

**Table 2:** Bone mineral density (BMD) at lumbar spine scores according to sex and age.

	*N*	Healthy (%)	Osteopenic (%)	Osteoporotic (%)	*T*-score lumbar spine
Male ≤ 30 y (range 18–30)	9	77.8	11.1	11.1	−0.9 ± 1.0
Male 31–50 y	9	33.3	55.6	11.1	−1.5 ± 0.9
Male > 50 y (range 51–70)	5	20	60	20	−1.0 ± 1.4
Female ≤ 30 y (range 17–30)	37	45.9	43.3	10.8	−1.1 ± 1.2
Female 31–50 y	78	50	39.8	10.2	−1.0 ± 1.1
Female > 50 y (range 52–75)	31	6.4	25.8	67.7	−2.6 ± 1.0

*N*: number.
